# A Survey of Frozen Phantom Limb Experiences: Are Experiences Compatible With Current Theories

**DOI:** 10.3389/fneur.2018.00599

**Published:** 2018-07-24

**Authors:** Kassondra L. Collins, Katherine E. Robinson-Freeman, Ellen O'Conor, Hannah G. Russell, Jack W. Tsao

**Affiliations:** ^1^Department of Anatomy and Neurobiology, University of Tennessee Health Science Center, Memphis, TN, United States; ^2^Department of Neurology, University of Tennessee Health Science Center, Memphis, TN, United States; ^3^Childrens Foundation Research Institute, Le Bonheur Children's Hospital, Memphis, TN, United States

**Keywords:** amputee, phantom limb pain, frozen limb, phantom limb sensation, proprioception

## Abstract

There are over two million individuals living with amputations in the United States. Almost all will experience the feeling of the amputated limb as still present, termed phantom limb sensation (PLS). Over 85% will also experience excruciatingly painful sensations known as phantom limb pain (PLP). Additionally some amputees also experience a sensation of the phantom limb in which the limb is immobile or stuck in a normal or abnormal anatomical position, termed frozen phantom sensations. When an amputee experiences a frozen limb they report that they are unable to move the limb, and sometimes report sensations of cramping and pain along with this immobility, fortunately not all frozen limbs are painful. Such sensations have previously been attributed to proprioceptive memories of the limb prior to amputation or a mismatch between visual feedback and proprioceptive feedback resulting from the initiation of a movement. Unfortunately there has been a dearth of research specifically focused on the frozen PLS. We conducted a survey to better elucidate and understand the characteristics and experiences of frozen PLSs. Results from the survey provided descriptions of a variety of frozen limb experiences, such as position and feelings experienced, combined with other phantom pain sensations, casting doubt on previous theories regarding frozen limbs. Further research needs to be focused on the etiology of phantom sensations and pain, which may not necessarily be maintained by the same processes, in order to understand better ways to treat PLP, increase mobility, and enhance amputees quality of life.

## Introduction

After the amputation of a limb, most amputees still feel that the limb is present. This experience is termed phantom limb sensation (PLS). More than 85% of amputees will also experience episodes of excruciating pain within the phantom limb, characterized by feelings of electric shocks, stabbing, and/or burning, which are termed phantom limb pain (PLP), a debilitating condition that drastically affects the well-being and daily quality of life. Additionally some amputees also experience a feeling as if the phantom limb is frozen and/or stuck in a specific anatomic position, which may or may not be accompanied by cramping or other painful sensations. Frozen phantom limbs without the accompanying pain are PLSs, however at times the feeling of a frozen limb may be painful as well. Further research is needed on the correlation between mobility of the phantom limb and quality of life of the amputees. Unfortunately the etiology of phantom limb experiences as a whole is not understood. PLSs and PLP may or may not be controlled by the same mechanisms, and whether the peripheral nervous system or the central plays more of a role is still undetermined.

In 1994 Ramachandran studied mirror box effects on an amputee who experienced a painful paralyzed phantom limb that mimicked the paralysis, which was, experienced a year prior to the actual amputation. The previous theory behind such an experience stemmed from the idea that the brain had learned the paralysis, through visual and proprioceptive feedback, that the limb was in fact not following the desired commands while still intact (1). Ramachandran then reported in 1998 that phantom limbs, in patients he has seen, tend to be moveable shortly after an amputation, but become frozen or stuck in one position over time (2). Some patients seen by Ramachandran also experienced painful spasms in which a fist became tightly clenched and painful. It was these painful experiences that Ramachandran aimed to alleviate using his mirror box therapy (2). Reilly et al. also made a similar observation in 2006, noting that the range of motion and number of movements that an amputee could complete with the phantom limb decreased with increasing time since the amputation, often leaving the limb completely frozen (3). This information was reported as additional information noted by researchers in the studies and not the sole purpose to the research, which draws on the necessity of a study that explicitly explores the experience of frozen phantom limbs. This preliminary survey has been conducted to determine if these additional reports on frozen limbs capture the experiences of amputees.

The etiology of the frozen phantom has been proposed to be a lack of feedback from vision and proprioception when movement commands are initiated (2). An expanded hypothesis put forward a suggestion that a frozen phantom limb was a result of the brain storing the last known position of the limb in a “proprioceptive memory bank” (4). These two theories play off of one another in the sense that the brain “remembered” or “learned” the immobility of the limb possibly even prior to amputation. Methods to reduce PLP, and mobilize the frozen limb (when such a sensation causes pain) have been sought, with mirror therapy found to be the most promising (1, 2, 5–7). Mirror therapy involves the amputee viewing the reflected image of the intact limb in a mirror, and moving both the intact and phantom limbs at the same pace, leading to eventual reduction in PLP and movement of a frozen limb. Ramachandran's original description of mirror therapy described four of five amputees with a frozen limb stuck in a painfully clenched fist position who had pain relief when they were able to view the reflected image in a mirror (2). This study however was focused on mirror therapy and its ability to induce movement in a phantom limb, and diminish PLP, not specifically looking at frozen phantoms. There have been no studies to date specifically focused on examining the occurrence and experiences of the immobile PLS, and only a few studies have even mentioned the phenomenon within their other findings. The current study is a detailed survey of 17 amputees who specifically experience a frozen phantom limb.

## Materials and methods

### Survey

The Institutional Review Board at the University of Tennessee Health Science Center, Memphis, TN gave approval for the study. The survey was conducted via telephone, all participants were required to listen to a consent statement and verbally consent to participation prior to administration of the survey to examine the nature of frozen PLSs. The survey queried demographics, the cause of amputation, the presence or absence of any pain or paralysis prior to the amputation, and a description of any PLP and/or PLS, how often they occurred and if they used any therapies to treat their painful experiences. Once the background information was collected, data regarding the specific frozen phantom limb was collected, including the frequency of experiences, the position(s) of the frozen limb, and any associated sensations. A research team member contacted each participant and the survey was given over the phone. This method insured that the research member could adequately present the question and query the participant further if more information was needed to answer. By conducting the survey in this manner the team was able to provide the participants with explanations of the various types of phantom experiences and make sure they understood differences between them.

### Participants

Amputees who participated in a separate research study and who expressed interest in participating in future research were contacted to participate. Inclusion criteria included the experience of an immobile phantom limb (frozen limb) and willingness to participate in the survey. Exclusion criteria included never experiencing a frozen phantom limb. One hundred sixty five amputees were contacted to participate, of those who responded, only 17 experienced a frozen phantom limb.

## Results

There were 17 participants with a mean age of 49.8 ± 12.8 years (range: 34–69), of whom 15 were Caucasians and 2 were African Americans. Nine amputees (52.9 %) had their amputation between 1 and 5 years ago with the rest having their amputation more than 5 years ago. Two of the amputees surveyed were upper extremity amputees and 15 were lower extremity amputees. Ten participants lost their limb due to trauma while seven lost the limb due to disease related complications.

Seven amputees reported experiencing the frozen limb sensation on a daily basis (with one having all frozen sensations disappear after a year), three amputees experienced the sensation weekly, six amputees had a frozen limb monthly, and one had a single yearly episode. Obtaining reports on the frequency of frozen limbs was conducted to compare these experiences to previous reports that state that frozen limbs increase with time since the amputation (2, 3).

The two upper extremity amputees both reported that their frozen limb felt as if the arm was bent across the stomach, with one reporting that the arm was sticking from the side into the stomach, however if they lay down the arm feels as if it is still bent but sticking straight up. Four of the lower extremity amputees reported that when the frozen limb occurred it felt as if the calf muscles were painfully cramping. Additionally the foot felt as if it was sticking straight out, the foot was cramped, the toes were pointing to the ceiling, or the foot was stuck in a plantar flexion position. Five other amputees reported the frozen sensation involving the toes of the phantom limb. Specifically the toes were stuck in a curled position (2 reports, one with the foot facing downward), the toes and arch of the foot were painfully cramped, the toes were bent at unusual angles, or the toes were crossed over one another. When participants discussed the feeling of their toes bent at unusual angles this meant unnatural positions of the toes, such as sticking out to the side or straight up in the air when the foot was planted on the ground. Two participants reported that their phantom limb felt as if the leg was bent at the knee as if they were sitting in a chair, even while standing. One report documented the ankle being frozen, with the last three involving the position of the foot. Two reported the foot was turned inwards at a 90 degree angle, one with the toes splayed open, and the third reported the foot sticking straight up in the air.

In addition to the feeling that the phantom limb was frozen in one location, the amputees also reported additional sensations. In total 13 amputees experienced cramping sensations along with the immobility of the limb. Six amputees experienced throbbing, 11 experienced a tingling sensation, two felt electric shocks accompanying the frozen limb, one felt a stabbing sensation, and one felt numb.

Another sensation that has been reported by some amputees is the feeling that the limb has telescoped, or become shorter than the intact limb. Three amputees experienced this sensation. One amputee with the feeling that the foot was turned at an angle reported the foot being closer to the residual limb than the intact foot, the amputee whose toes were bent at unusual angles also reported that they were closer to the stump than they should be. The final amputee that experienced the frozen limb to be telescoped was the individual with the sensation of the foot sticking straight up in the air, it was reported to be attached to the end of the residual limb and not where the foot belongs.

**Figure 1:**
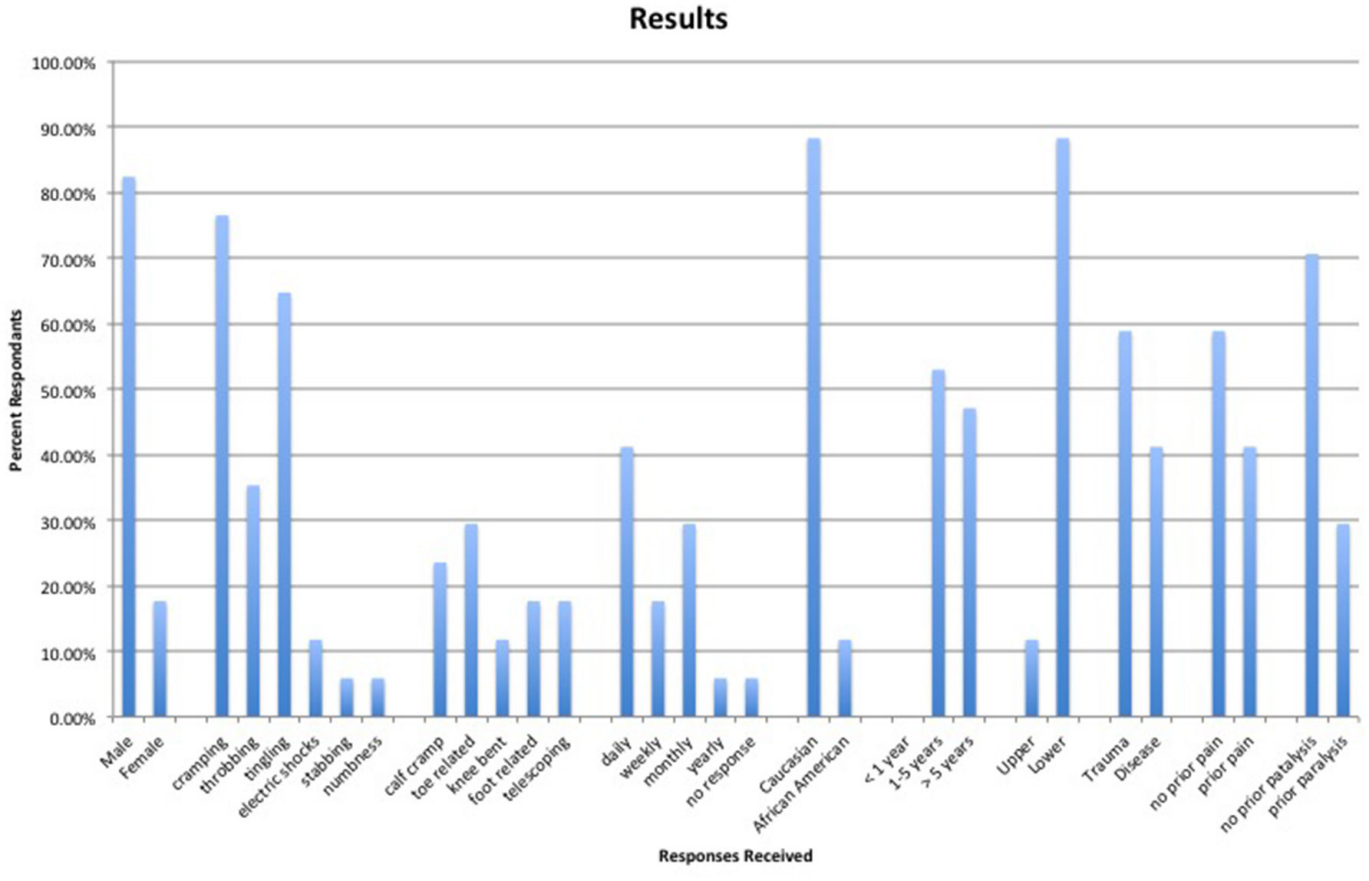
All responses visually reproduced.

To compare our survey responses to previous research and hypotheses we investigated the prevalence of pain and paralysis prior to amputation. Ten amputees reported that they either did not have pain prior to the amputation or that the pain experienced prior to the amputation was not the same as the phantom pain experienced. Seven amputees did experience pain before the amputation that was similar to the phantom pain. Although these numbers seem to directly correlate with the disease vs. trauma amputations, one amputee who had disease related complications did not experience pain prior to the amputation. Additionally, out of the 17 amputees with frozen limb sensations, 12 did not experience any paralysis prior to the amputation. Five however did experience some paralysis prior to the amputation. All responses gathered are visually reproduced in Figure 1.

When questioned about the use of therapies to treat the PLP sensations, survey respondents were discouraged by the lack of pain relief provided. Many amputees had tried combinations of mirror therapy, medication, and stimulation therapies in attempts to diminish their pain experiences. Eight amputees attempted mirror therapy, 12 tried medications at some point, and seven used stimulation therapies. Of those who reported using mirror therapy, they stated that the therapy was specifically to help reduce pain, not to increase mobility, although more movement may have assisted with the pain, however this has not been directly studied. None of the amputees who tried mirror therapy were currently still using the therapy. Reports included that mirror therapy worked until there were changes in temperature, helped a little bit, reduced some pain, didn't work at all, and even caused more pain due to increased thoughts about the phantom limb.

Medications used to attempt to control PLP included; Aleve, Gabapentin, Arnica (herbal), Lyrica, and an unnamed sodium channel blocker. Gabapentin was reported to work the best at relieving painful sensations, not increasing mobility of a frozen limb, however most reports stated that is only relieved the pain temporarily. Other reports stated that no medication worked, and that the side effects were worse than the PLP. All medications were prescribed in order to diminish the PLP not increase mobility in the phantom. Stimulation therapies reported included massage and transcutaneous electrical nerve stimulation (TENS). Reports stated that these therapies worked a little to temporarily reduce pain, yet were not sufficient therapies.

## Discussion

This survey found that those who experience a frozen phantom limb do so at a varying rate, with some reporting daily frozen limb experiences while others experience it more sporadically. Our results are in contrast to previous research suggesting that immobile limbs become more prominent over time and that the ability to move the phantom diminishes with time (2, 3). Out of the participants who had an amputation more than 5 years ago, the most frequent report of a frozen limb was one time a week. The majority of frequencies were once every month. There were also two reports of no frozen sensations for more than 10 years in those amputees who had a limb removed more than 5 years ago.

Additionally one amputee who experienced frozen limb sensations daily had these experiences resolve after 1 year, also contrary to previous research. Our results also suggest that frozen phantom limbs are not likely due to a learned paralysis. The majority (70.6%) of our amputees surveyed reported experiencing frozen limbs with no paralysis prior to amputation. Furthermore, we found that the frozen phantom limb does not always assume the position of the last memory of the limb for the majority (60%) of amputees who did experience paralysis prior to amputation. Only five amputees reported that their limb was paralyzed prior to amputation, with two reporting a similar frozen limb to that experience. In addition less than half of the amputees experienced PLP that was similar to pain experienced before the limb was removed. Although the sample size of our survey was smalls, the results indicate that additional research should be directed toward elucidating the causes of PLS, PLP, and why some amputees experience the sensation of a frozen limb, both painful and non-painful. Even with the small number of 17 participants the survey shows that the experiences reported do not line up with previous hypotheses regarding frozen phantom limbs. With this preliminary information future research needs to be conducted using larger sample sizes. Another interesting route of research would be whether similarities exist between the frozen phantom limb and freezing experienced in other neurological disorders, such as with stroke patients, and/or Parkinson disease gait freezing.

This survey expressed the fact that none of the available therapies to treat PLP and frozen phantom limbs work to eliminate such experiences in every amputee. The general consensus, from those who participated, was that mirror therapy, medication, and stimulation therapies worked to alleviate some pain temporarily, at best. In the case of mirror therapy, it is possible that participants were not completing the therapy in the correct manner, or not sticking with the treatment long enough for effects to be observed. Little is known regarding the best practice measures for applying mirror therapy and therefore methods of practice vary drastically (8). Without understanding the etiology of such phantom phenomenon it is hard to prescribe a medication that directly targets the pain, and there are no medications to induce movement of a phantom limb. Of the medications mentioned amputees were taking an NSAID, calcium channel blocker, sodium channel blocker, GABA analogue, and topical skin treatments. Through this list alone it is clear that the mechanisms of action for PLP relief are in drastic need of further research. The current literature does not provide us with research that specifically investigates the experience of the frozen phantom limb, whether it be painful or non-painful. Our study was the first of its kind to question amputees specifically on frozen limb experiences. It was a very small study but will instill interest and expansion on research into the topic. Understanding the pathways that cause PLS and PLP may provide us with more information regarding the experience of a frozen phantom limb, which can be painful or non-painful. Research may need to begin to focus on the phantom phenomenon as separate entities that each need to be studied to further understand how to minimize pain, enhance function, and therefore provide a better quality of life for amputees.

## Author contributions

KC conducted surveys, analyzed the data, and drafted the manuscript. KR-F assisted with data analysis, created figures, and edited the manuscript. EO assisted with statistical analysis and figure creation. HR conducted surveys. JT oversaw study execution and manuscript editing.

### Conflict of interest statement

The authors declare that the research was conducted in the absence of any commercial or financial relationships that could be construed as a potential conflict of interest.

## References

[1] Ramachandran VS. (1994). Phantom limbs, neglect syndromes, repressed memories, and Freudian psychology. Int Rev Neurobiol. 37:291–333: discussion 369–72. 7883483 10.1016/s0074-7742(08)60254-8

[2] Ramachandran VS. Consciousness and body image: lessons from phantom limbs, Capgras syndrome and pain asymbolia. Philos Trans R Soc Lond B Biol Sci. (1998) 353:1851–9. 10.1098/rstb.1998.0337 9854257 PMC1692421

[3] Reilly KT Mercier C Schieber MH Sirigu A. Persistent hand motor commands in the amputees' brain. Brain (2006) 129:2211–23. 10.1093/brain/awl154 16799174

[4] Anderson-Barnes VC McAuliffe C Swanberg KM Tsao JW. Phantom limb pain–A phenomenon of proprioceptive memory? Med Hypotheses (2009) 73:555–8. 10.1016/j.mehy.2009.05.038 19556069

[5] Chan BL Witt R Charrow AP Magee A Howard R Pasquina PF. Mirror therapy for phantom limb pain. N Engl J Med. (2007) 357:2206–7. 10.1056/NEJMc071927 18032777

[6] Foell J Bekrater-Bodmann R Diers M Flor H. Mirror therapy for phantom limb pain: brain changes and the role of body representation. Eur J Pain (2014) 18:729–39. 10.1002/j.1532-2149.2013.00433.x 24327313

[7] Finn SB Perry BN Clasing JE Walters LS Jarzombek SL Curran S. A randomized, controlled trial of mirror therapy for upper extremity phantom limb pain in male amputees. Front Neurol. (2017) 8:267. 10.3389/fneur.2017.00267 28736545 PMC5500638

[8] Rothgangel AS Braun SM Beurskens AJ Seitz RJ Wade DT. The clinical aspects of mirror therapy in rehabilitation: a systematic reviewsref-list of the literature. Int J Rehabil Res. (2011) 34:1–13. 10.1097/MRR.0b013e3283441e98 21326041

